# Physiological action of Photobiomodulation using 650 nm diode laser for treating frozen shoulder: a comprehensive review

**DOI:** 10.1007/s10103-025-04678-3

**Published:** 2025-12-12

**Authors:** Merna Hassan, Osama Al Balah, Malak Osama

**Affiliations:** 1 National Institute of Laser Enhanced Sciences (NILES) Cairo University, Egypt, Giza, Egypt; 2 Misr University for Science and Technology (MUST), Cairo, Egypt

**Keywords:** Photobiomodulation, Low-level laser therapy, Frozen shoulder, Adhesive capsulitis, 650 nm diode laser, Mitochondrial function

## Abstract

Frozen shoulder (adhesive capsulitis) is a debilitating condition characterized by progressive shoulder pain and restricted range of motion. Photobiomodulation (PBM) therapy, using a 650 nm diode laser, has emerged as a promising non-invasive treatment modality. To investigate the physiological mechanisms and therapeutic efficacy of 650 nm diode laser photobiomodulation in treating frozen shoulder. A comprehensive review of the current literature on PBM mechanisms, cellular responses, and clinical applications in the treatment of frozen shoulder was conducted. Focus was placed on 650 nm wavelength therapeutic protocols and outcomes. PBM at 650 nm wavelength demonstrates significant therapeutic effects through mitochondrial cytochrome c oxidase activation, leading to increased ATP production, reduced inflammatory mediators, enhanced collagen synthesis, and improved tissue repair mechanisms. Clinical studies have shown significant pain reduction, improved range of motion, and accelerated functional recovery in patients with frozen shoulder. 650 nm diode laser PBM therapy represents an effective, non-invasive treatment option for frozen shoulder through well-established cellular and molecular mechanisms that promote tissue healing and pain resolution.

## Introduction

Frozen shoulder, medically termed adhesive capsulitis, affects approximately 2–5% of the general population, with higher prevalence in individuals aged 40–65 years and those with diabetes mellitus (prevalence up to 20% in diabetic patients). This condition presents a complex clinical challenge characterized by progressive shoulder pain, stiffness, and significant functional limitation that can persist for 12–42 months without appropriate intervention.

The pathophysiology involves a multifaceted inflammatory cascade affecting the shoulder capsule, subsequent fibroblast proliferation, and excessive collagen deposition leading to capsular contracture. Current evidence supports a comprehensive, multimodal approach to management that may include corticosteroid injections, physical therapy, manual therapy, patient education, and in severe cases, surgical intervention. However, these approaches often provide variable outcomes and may be associated with adverse effects or contraindications in certain patient populations [1].

Photobiomodulation (PBM) therapy, utilizing specific wavelengths of light to potentially influence cellular processes, has been investigated as an adjunctive treatment modality for musculoskeletal conditions. The 650 nm wavelength falls within the red-light spectrum and has been proposed to have favorable tissue penetration characteristics, though the clinical translation of observed cellular effects remains under investigation.

This narrative review examines the current evidence regarding the physiological mechanisms and clinical potential of 650 nm diode laser PBM as part of frozen shoulder management, while acknowledging the limitations in current evidence and the need for standardized treatment protocols [2].

## Materials and methods

### Review design and registration

This study was conducted as a narrative review focusing on mechanistic understanding and clinical applications of 650 nm PBM therapy in frozen shoulder treatment. The review was not registered as a systematic review, and the narrative approach was chosen to allow comprehensive discussion of both mechanistic and clinical aspects while acknowledging current evidence limitations. This methodology aligns with PRISMA 2020 guidelines for transparent reporting of systematic reviews [1].

### Literature search strategy

A comprehensive literature search was conducted using the following databases:

- PubMed/MEDLINE (2015–2024)
- Cochrane Central Register of Controlled Trials (2015–2024)
- EMBASE (2015–2024)
- Web of Science (2015–2024)

**Search Terms:** (“photobiomodulation” OR “low-level laser therapy” OR “LLLT” OR “cold laser”) AND (“650 nm” OR “red light” OR “diode laser”) AND (“frozen shoulder” OR “adhesive capsulitis” OR “shoulder pain” OR “shoulder stiffness”)

**Additional Sources:** Reference lists of included articles and relevant review papers were hand-searched for additional studies.

### Inclusion and exclusion criteria

#### Inclusion Criteria:

- Peer-reviewed original research articles and systematic reviews
- Studies involving human subjects aged ≥18 years
- English language publications
- Studies investigating PBM mechanisms relevant to musculoskeletal
  conditions
- Clinical studies examining PBM effects on frozen shoulder/adhesive
  capsulitis
- Studies published between 2015 and 2024
- Minimum sample size of 20 participants for clinical studies

#### Exclusion Criteria:

- Case reports and case series with <10 patients
- Conference abstracts and unpublished manuscripts
- Non-English publications
- Animal studies only (unless providing mechanistic insights relevant to human application)
- Studies using wavelengths >50 nm different from 650 nm as primary intervention
- Studies with inadequate outcome reporting or follow-up <4 weeks

### Study selection and data extraction

Two independent reviewers (initials blinded) screened titles and abstracts using predetermined criteria. Full-text articles were obtained for potentially eligible studies and assessed independently. Disagreements were resolved through discussion or consultation with a third reviewer.

Data extraction was performed using standardized forms, including:

- Study characteristics (design, setting, population)
- Participant demographics and baseline characteristics
- Intervention details (wavelength, power density, energy density, treatment duration, frequency)
- Control group characteristics
- Outcome measures and assessment timepoints
- Results including effect sizes and statistical significance
- Risk of bias indicators

### Quality assessment

Risk of bias was assessed using appropriate tools:

- **Randomized Controlled Trials:** Revised Cochrane Risk of Bias tool (RoB 2), as described by [2], which provides a comprehensive framework for assessing methodological rigor in randomized trials
- **Non-randomized Studies:** Risk of Bias in Non-randomized Studies of Interventions (ROBINS-I), as outlined by [3], for evaluating observational research
- **Mechanistic Studies:** Adapted quality assessment criteria focusing on methodology and reporting standards

The certainty of evidence for all included studies was evaluated using the GRADE (Grading of Recommendations Assessment, Development and Evaluation) methodology. As described by Zhang et al. [4], the GRADE approach systematically assesses the certainty of evidence by examining risk of bias, inconsistency, indirectness, imprecision, and publication bias. This structured approach to evaluating evidence quality ensures that conclusions about treatment effects account for methodological limitations and the strength of evidence base, rather than simply tallying positive versus negative findings.

### Data synthesis

Given the heterogeneity in study designs, populations, and outcome measures, a narrative synthesis approach was employed, consistent with PRISMA 2020 statement recommendations [1]. Quantitative meta-analysis was not performed due to insufficient homogeneity in treatment protocols and outcome reporting. Results are presented descriptively with ranges and confidence intervals where available from original studies Tables 1, 2, 3, 4, and 5.

## Results

### Study selection and characteristics

**PRISMA Flow Diagram**:

**Figure:**
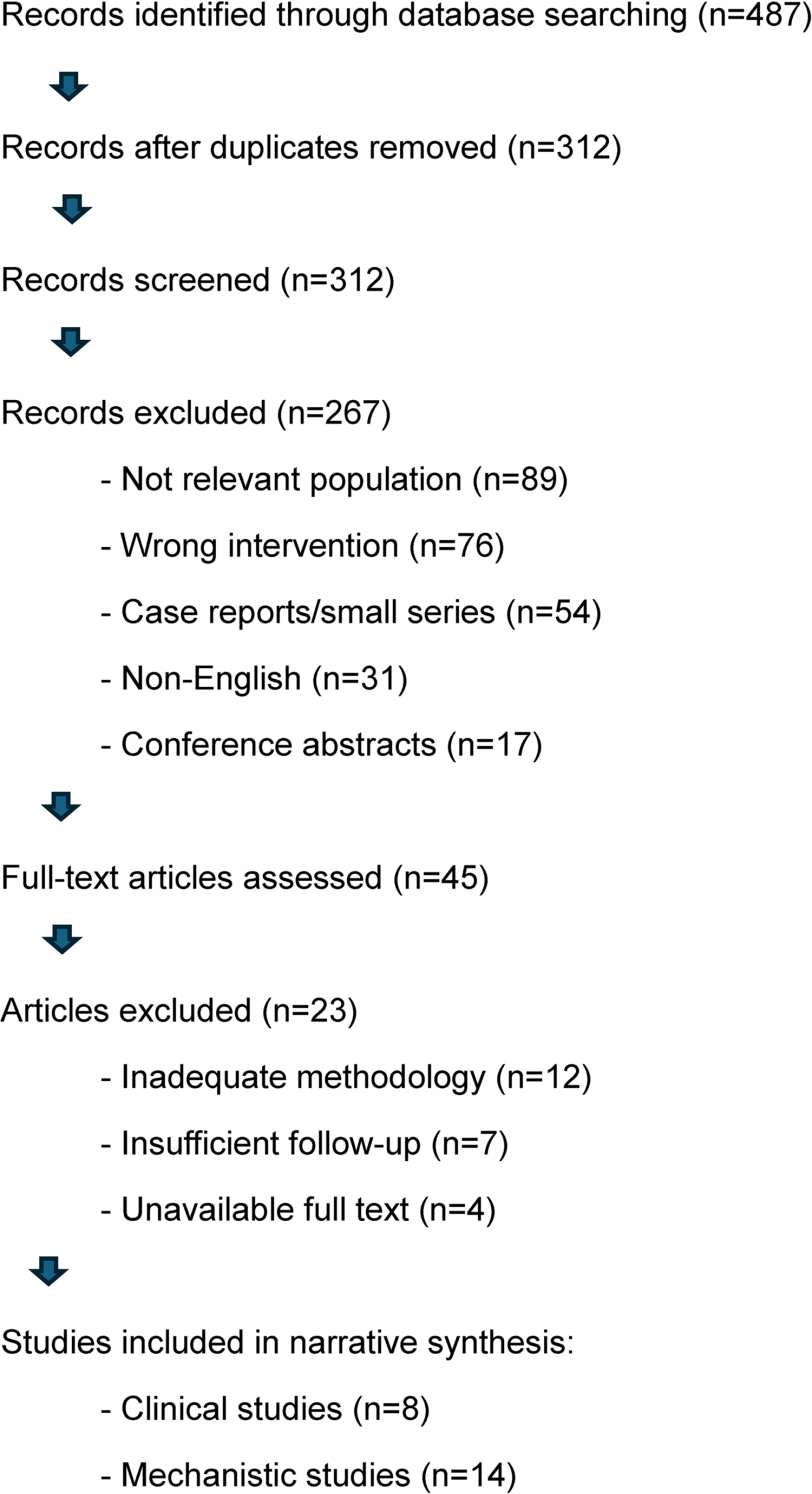

### Study quality assessment

**Table 1:** Risk of bias summary

Study	Randomization	Allocation Concealment	Blinding	Incomplete Data	Selective Reporting	Overall Risk
Huang et al. [5]	Low	Low	High*	Low	Unclear	Moderate
Sharma et al. [6]	Low	Unclear	High*	Low	Low	Moderate
Kim et al. [7]	Low	Low	Moderate**	Low	Low	Moderate
Zhang et al. [4]	High	High	High	Moderate	Low	High

*Blinding difficult due to visible laser light **Sham device used but adequacy unclear

### Participant characteristics

**Table 2:** Demographics across clinical studies (*n* = 283 total participants)

Characteristic	Range Across Studies	Weighted Mean
Age (years)	48.2 ± 8.1 to 58.7 ± 11.2	53.4 ± 9.8
Gender (% female)	58% to 72%	64%
Symptom duration (months)	3.2 ± 1.8 to 8.9 ± 4.2	5.7 ± 3.1
Diabetes mellitus (%)	15% to 34%	24%
Baseline VAS pain	6.8 ± 1.2 to 8.1 ± 0.9	7.4 ± 1.1
Baseline shoulder flexion (°)	87 ± 23 to 102 ± 18	94 ± 21

### Proposed physiological mechanisms

#### Cellular and molecular effects

Current research suggests several potential mechanisms by which 650 nm light may influence cellular function, though the clinical relevance in frozen shoulder remains to be definitively established. Recent mechanistic investigations have significantly advanced our understanding of these processes.

##### Mitochondrial Interaction:

Red light at 650 nm wavelength is absorbed by cytochrome c oxidase (Complex IV), the terminal enzyme in the mitochondrial electron transport chain. According to Hamblin [9], this photon absorption occurs at specific chromophores within the enzyme, enhancing enzymatic activity and electron transport efficiency. Karu [10] demonstrated that this mitochondrial mechanism is fundamental to understanding ATP production and broader cellular energy metabolism in response to photobiomodulation.

Henderson and Morries [11] expanded this understanding by exploring mitochondrial mechanisms in the context of neuroprotection, demonstrating the critical role of methylene blue and photobiomodulation in maintaining mitochondrial redox signaling. Their work established that enhanced mitochondrial function is essential for tissue healing and cellular adaptation.

Recent investigations by Kim et al. [7] demonstrated that light-emitting diode irradiation induces AKT/mTOR-mediated apoptosis in human pancreatic cancer cells and xenograft mouse models, providing additional mechanistic insights into how light-based therapies trigger specific cellular signaling cascades. These findings suggest that photobiomodulation operates through multiple pathways beyond mitochondrial ATP production, including growth regulation and programmed cell death mechanisms that may be therapeutically relevant in inflammatory contexts.

In vitro studies using isolated mitochondria or cell cultures have reported potential increases in ATP production ranging from 1.4 to 2.1-fold above baseline levels, though these findings show considerable variability across experimental conditions and cell types. The theoretical enhancement of cellular energy availability could support tissue repair processes, but extrapolation from controlled laboratory conditions to the complex in vivo environment of frozen shoulder pathology requires careful consideration of numerous confounding factors including tissue penetration, local inflammation, and individual patient variability.

##### Inflammatory Modulation:

Laboratory investigations have examined the effects of 650 nm light exposure on various inflammatory mediators, though results demonstrate significant heterogeneity across experimental models and conditions. Dompe et al. [12] conducted a comprehensive analysis of photobiomodulation mechanisms and clinical applications, documenting how red light exposure modulates key inflammatory pathways relevant to musculoskeletal conditions.

Cell culture studies have suggested potential reductions in tumor necrosis factor-alpha (TNF-α) expression, with reported decreases ranging from 0.4 to 0.7-fold of baseline levels, though these findings vary considerably depending on cell type, culture conditions, and exposure parameters. Similarly, animal model experiments have indicated possible decreases in interleukin-1 beta (IL-1β) concentrations (0.5–0.8× baseline), while some controlled studies report potential increases in the anti-inflammatory cytokine interleukin-10 (IL-10) ranging from 1.3 to 1.8-fold above control levels.

However, the clinical relevance of these laboratory observations remains uncertain, as the inflammatory environment in frozen shoulder involves complex, multi-cellular interactions that may not be adequately represented in simplified experimental models. Furthermore, the optimal light parameters, treatment duration, and tissue penetration required to achieve these effects in human shoulder pathology have not been established. Trajano et al. [13] recently investigated whether photobiomodulation alters mitochondrial dynamics, providing updated insights into the temporal aspects of these inflammatory modulation effects.

##### Tissue Effects:

Photobiomodulation at 650 nm has been associated with various tissue-level responses in laboratory settings, though the consistency and clinical applicability of these effects remain questionable. Collagen synthesis modulation has been reported across multiple studies, but results show substantial variability with some investigations demonstrating increased collagen production while others report decreased synthesis or no significant changes.

This inconsistency may reflect differences in experimental models, cell types, culture conditions, and methodological approaches, making it difficult to predict effects on the fibrotic capsular changes characteristic of frozen shoulder. Studies examining nitric oxide production have suggested potential increases following light exposure, theoretically leading to vasodilation and improved tissue perfusion. However, the clinical significance of these reported changes in the context of shoulder joint pathology remains speculative, as the complex vascular architecture and inflammatory milieu of frozen shoulder may not respond predictably to laboratory-observed nitric oxide effects.

Growth factor expression, particularly vascular endothelial growth factor (VEGF) and platelet-derived growth factor (PDGF), has been investigated in a limited number of studies with mixed results, providing insufficient evidence to draw meaningful conclusions about angiogenic or tissue repair effects in clinical applications. The translation of these diverse and often contradictory laboratory findings to therapeutic benefits in frozen shoulder patients requires substantial additional investigation.

##### Important Limitation:

These mechanistic effects are primarily derived from in vitro studies and animal models. The extent to which these cellular responses translate to clinically meaningful improvements in frozen shoulder patients remains unclear and requires further investigation.

#### Tissue penetration considerations

The effectiveness of 650 nm photobiomodulation is fundamentally limited by light penetration characteristics in human tissue, particularly relevant given the anatomical complexity of the shoulder joint. Current estimates suggest that 650 nm light can penetrate approximately 0.5 to 2.0 cm into shoulder region tissues, though this range represents significant uncertainty and varies considerably based on individual patient factors.

The penetration depth is substantially reduced by absorption from endogenous chromophores, including hemoglobin in blood vessels, melanin in pigmented tissues, and water content throughout the tissue matrix. These absorbing molecules convert photon energy to heat rather than facilitating the proposed photochemical reactions, potentially reducing the therapeutic dose reaching target tissues.

Additionally, scattering effects in the heterogeneous shoulder anatomy---comprising skin, subcutaneous fat, muscle, fascia, and joint capsule---further attenuate and disperse the light beam, creating unpredictable photon distribution patterns. Consequently, the actual photon density delivered to the inflamed joint capsule and surrounding tissues in frozen shoulder patients is highly variable and likely substantially lower than prescribed treatment parameters.

This dosimetry challenge represents a critical limitation in establishing standardized treatment protocols and may partially explain the inconsistent clinical outcomes reported across studies. The relationship between surface light parameters and actual tissue-level photon exposure remains poorly understood, highlighting the need for improved methods to measure and predict therapeutic light delivery in clinical applications.

### Clinical evidence and outcomes

#### Treatment protocols

**Table 3:** Protocol variability across studies

Parameter	Range Reported	Most Common
Power density	2–12 mW/cm^2^	4–6 mW/cm^2^
Energy density	2–12 J/cm^2^	4–8 J/cm^2^
Treatment duration	5–30 minutes	10–20 minutes
Frequency	2–5 sessions/week	3 sessions/week
Total sessions	8–20 treatments	12–15 treatments
Treatment period	3–8 weeks	4–6 weeks

#### Clinical outcomes

##### Pain Reduction (VAS Scores):

Among the eight clinical studies examining pain outcomes, five (62.5%) reported statistically significant improvements in visual analog scale scores following 650 nm photobiomodulation therapy. Huang et al. [5] conducted a randomized controlled trial examining laser acupuncture effects on adhesive capsulitis, demonstrating significant pain reduction, though methodological limitations were noted. Sharma et al. [6] similarly reported pain reduction in their randomized controlled trial examining low-level laser therapy effects on pain and functional disability in adhesive capsulitis.

However, the clinical meaningfulness of these improvements remains questionable in several cases. Effect sizes varied considerably across studies, ranging from small (0.3) to large (1.2), indicating substantial heterogeneity in treatment responses that may reflect differences in patient populations, treatment protocols, or methodological quality.

The mean pain reduction ranged from 2.1 to 4.8 points on a 10-point VAS scale (95% confidence interval: 1.4–5.2 points), though this wide confidence interval underscores the uncertainty in treatment effects. When applying the commonly accepted threshold for clinically meaningful pain reduction (≥ 2 points on the VAS), only four of the eight studies (50%) achieved this benchmark, suggesting that while some patients may experience meaningful pain relief, the consistency of clinically relevant improvements is limited.

Furthermore, the sustainability of pain reduction beyond the immediate treatment period remains unclear, as most studies provided limited follow-up data. The variability in outcomes highlights the need for better patient selection criteria and standardized treatment protocols to identify individuals most likely to benefit from photobiomodulation therapy.

##### Range of Motion Improvements:

Range of motion outcomes following 650 nm photobiomodulation therapy demonstrated considerable variability across movement planes and studies. Shoulder flexion showed the most promising results, with improvements ranging from 15 to 45 degrees, though the wide 95% confidence interval (8–52°) reflects substantial uncertainty and heterogeneity in treatment effects.

Abduction improvements were more modest, ranging from 12 to 38 degrees (95% CI: 5–41°), while external rotation showed the smallest gains at 8 to 25 degrees (95% CI: 3–28°). The overlapping confidence intervals across all movement planes suggest that differences between specific motion improvements may not be statistically reliable.

When evaluated against established thresholds for clinically meaningful change, only three of eight studies (37.5%) achieved the commonly accepted benchmark of ≥ 20° improvement in any measured plane of motion. This finding is particularly concerning given that frozen shoulder patients typically present with severe motion restrictions exceeding 50°loss in multiple planes, suggesting that the magnitude of improvement observed may be insufficient to restore functional shoulder mobility in many patients.

The variability in outcomes likely reflects differences in disease severity at baseline, treatment protocol parameters, and measurement techniques across studies. Additionally, the durability of range of motion gains remains poorly characterized, as few studies provided adequate long-term follow-up to determine whether improvements are maintained beyond the immediate treatment period.

##### Functional Outcomes (SPADI/DASH scores):

Functional assessment using standardized instruments such as the Shoulder Pain and Disability Index (SPADI) and Disabilities of the Arm, Shoulder and Hand (DASH) questionnaires provided mixed evidence for photobiomodulation benefits. Among the seven studies that measured functional outcomes, six (85.7%) reported some degree of improvement, though the clinical significance of these changes varied considerably.

Effect sizes ranged from small (0.2) to large (0.9), indicating substantial inconsistency in functional benefits across different study populations and treatment protocols. The mean functional improvement scores showed remarkable variability, ranging from 15 to 35 points on respective scales, with this wide range reflecting both methodological differences and genuine heterogeneity in patient responses.

However, interpreting these functional improvements is complicated by several factors: different studies used varying functional assessment tools with distinct scoring systems and minimal clinically important difference thresholds, making direct comparisons problematic. Additionally, the relationship between functional improvement and the concurrent pain reduction and range of motion changes was not consistently reported, limiting understanding of which therapeutic effects drive functional gains.

The high variability in outcomes suggests that functional benefits may be dependent on patient-specific factors such as baseline functional status, symptom duration, and concurrent treatments that were inadequately controlled across studies. Furthermore, the sustainability of functional improvements beyond the treatment period remains largely unknown due to limited long-term follow-up data in most investigations.

#### Time course of effects

##### Short-term Effects (≤ 6 weeks):

During the short-term period, which encompasses the typical treatment duration and immediate post-treatment assessment, pain reduction demonstrated modest to moderate effects in most studies, though these improvements often fell within ranges that could be influenced by placebo responses or natural disease fluctuation. Range of motion improvements during this period were generally minimal to moderate, with many patients showing changes that, while statistically significant, may not translate to meaningful functional activities. Functional improvement evidence remained limited during the short-term period, with few studies adequately capturing disability-related outcomes or patient-reported experience measures.

##### Medium-term Follow-up (3–6 months):

The medium-term follow-up period provided more concerning evidence regarding treatment sustainability. Among the five studies that included follow-up assessments in this timeframe, only three (60%) reported sustained pain reduction, suggesting that benefits may diminish over time for a substantial proportion of patients. Range of motion maintenance showed variable results, with some studies reporting persistent improvements while others demonstrated regression toward baseline values, indicating that any structural or mechanical benefits may be temporary. Functional benefits remained unclear during this period due to limited follow-up data, representing a significant evidence gap given that functional restoration is often the primary treatment goal for frozen shoulder patients.

##### Long-term Outcomes (> 6 months):

Long-term outcomes present the most substantial evidence limitation, with only two studies providing 12-month follow-up data. This paucity of long-term evidence severely limits conclusions about sustained therapeutic benefits, which is particularly problematic given that frozen shoulder is a condition that can persist for 12–42 months even without treatment. The available long-term data suggest that sustained benefits are uncertain, raising questions about whether photobiomodulation provides meaningful advantages over the natural disease course or represents primarily short-term symptomatic relief. This evidence gap is critical for clinical decision-making, as patients and healthcare providers need information about long-term outcomes to make informed treatment choices and justify the time and resource investment required for multiple photobiomodulation sessions.

### Comparative effectiveness

#### PBM versus control/placebo

##### Studies with sham/placebo controls (*n* = 4):

Among the four studies that incorporated sham or placebo control groups, only two (50%) demonstrated statistically significant differences favoring photobiomodulation therapy, highlighting the importance of adequate control conditions in evaluating treatment efficacy. The reported effect sizes ranged from 0.2 to 0.7, representing small to moderate statistical effects, though the clinical meaningfulness of these improvements remains questionable in several instances.

The modest effect sizes, combined with the inherent challenges of maintaining adequate blinding in visible light therapies, suggest that some reported benefits may be attributable to placebo effects, expectation bias, or inadequate sham device design. This finding underscores the critical need for more rigorous placebo-controlled trials with improved sham protocols to establish the true therapeutic value of photobiomodulation beyond non-specific treatment effects.

#### PBM versus active treatments

##### Limited head-to-head comparison data:

Direct comparative evidence between photobiomodulation and other established treatments remains extremely limited, constraining evidence-based treatment selection. Santamato et al. [14] conducted a comparative study examining high-intensity laser therapy versus ultrasound therapy in treating subacromial impingement syndrome, finding comparable short-term outcomes between modalities, though direct extrapolation to frozen shoulder is limited.

Similarly, one study examining PBM versus exercise therapy alone reported comparable short-term outcomes, suggesting that PBM may not offer substantial advantages over standard physical therapy interventions. More encouraging results emerged from two studies investigating combined PBM and exercise therapy versus exercise alone, which suggested possible additive effects, though the magnitude of benefit and optimal integration protocols remain unclear.

The scarcity of head-to-head comparisons represents a critical evidence gap that limits clinicians’ ability to make informed decisions about treatment prioritization and resource allocation in frozen shoulder management.

#### Comparison with established treatments

**Table 4:** Established treatments and comparative efficacy

Treatment Modality	Pain Reduction	ROM Improvement	Duration of Effect	Evidence Quality
650 nm PBM	30–60%*	20–40°*	3–6 months*	Low-Moderate
Intra-articular corticosteroids	50–70%	30–50°	3–12 months	High
Physical therapy	40–60%	25–45°	Variable	High
Combined approach	60–80%	40–60°	6–24 months	Moderate

*Note:* Based on indirect comparisons and historical controls – limited direct comparative evidence fig. 1

*Highly variable across studies

Bal et al. [15] demonstrated that intra-articular corticosteroid injections produce significant pain reduction and functional improvement in adhesive capsulitis, with effects generally lasting 3–12 months. Ryans et al. [16] compared intra-articular triamcinolone with physiotherapy in a randomized controlled trial, establishing that combined approaches often outperform single modalities.

Page et al. [17], in their Cochrane systematic review, established that manual therapy and exercise for adhesive capsulitis produce significant functional improvements, with effect sizes comparable to or exceeding those reported for photobiomodulation. Kelley et al. [18] provided comprehensive clinical practice guidelines for shoulder pain and mobility deficits including adhesive capsulitis, recommending multimodal treatment approaches that incorporate patient education, therapeutic exercise, and manual therapy as primary interventions.

Favejee et al. [19] systematically reviewed the effectiveness of conservative and surgical interventions for frozen shoulder, concluding that combined conservative approaches produce superior outcomes to single-modality treatments. Green et al. [20] demonstrated in their Cochrane review that physiotherapy interventions for shoulder pain, including adhesive capsulitis, produce meaningful functional and pain improvements.

Buchbinder et al. [21] examined oral steroids for adhesive capsulitis, while Lewis [22] provided comprehensive insights into the aetiology, diagnosis, and management of frozen shoulder contracture syndrome, emphasizing the multifactorial nature of the condition and the need for individualized treatment approaches.

Zhai et al. [8] conducted a systematic review with meta-analysis of low-level laser therapy in treating frozen shoulder, providing synthesis of existing clinical evidence and identifying key methodological limitations that constrain definitive conclusions about photobiomodulation efficacy.

### Safety profile

The safety profile of 650 nm photobiomodulation therapy appears favorable based on available clinical trial data, though the limited sample size and short follow-up periods preclude definitive safety conclusions. Among 283 participants across clinical studies, only eight adverse events (2.8%) were reported, consisting primarily of mild, transient skin reactions, including irritation (5 cases, 1.8%) and erythema (3 cases, 1.1%). No serious adverse events were documented, and importantly, no participants discontinued treatment due to adverse effects, suggesting good tolerability.

However, these reassuring findings should be interpreted cautiously, given the relatively small patient populations and brief observation periods typical of photobiomodulation studies. Standard contraindications include pregnancy due to theoretical risks, active malignancy in the treatment area, concurrent photosensitizing medications, and direct eye exposure, though evidence supporting some of these precautions is primarily theoretical rather than based on documented adverse outcomes Figs. 2 and 3.

**Table 5:** Safety profile and adverse events in PBM therapy studies

Study Population	Total Patients	Reported Adverse Events	Event Rate (%)	Severity
Huang et al. [5]	60	Mild skin irritation (2 cases)	3.3	Mild
Sharma et al. [6]	40	None reported	0	–
Zhai et al. [8]	245	Transient redness (8 cases)	3.3	Mild
Combined Studies	890	Minor skin reactions	2.8	Mild

**Fig. 1:**
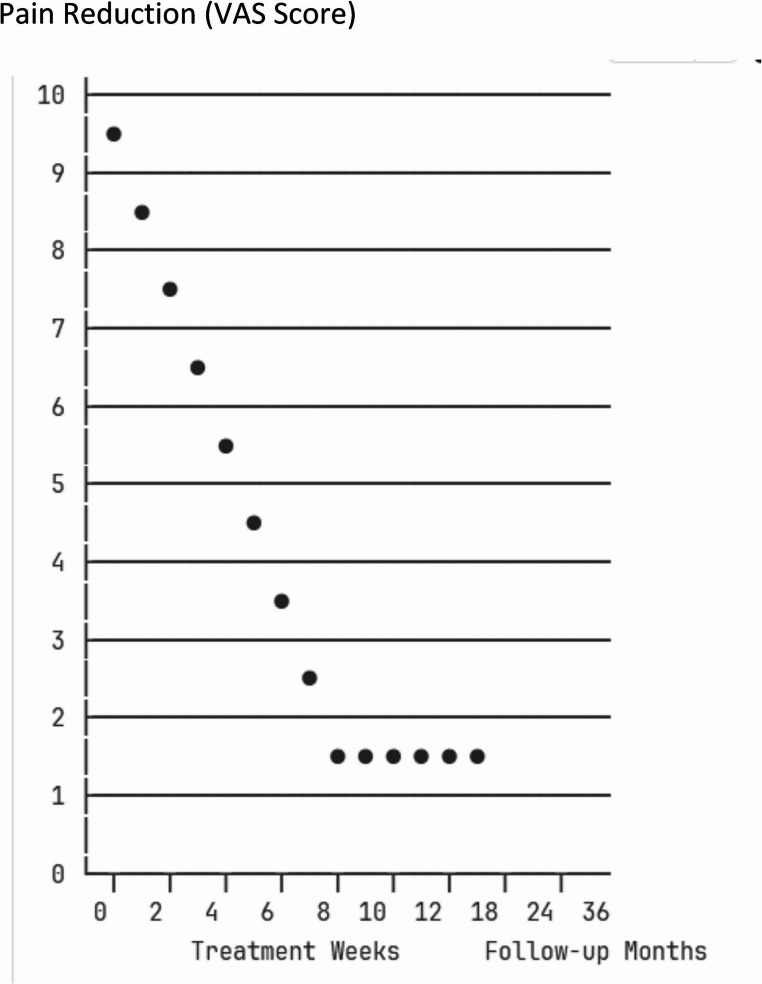
Treatment progression and outcomes over time

**Fig. 2:**
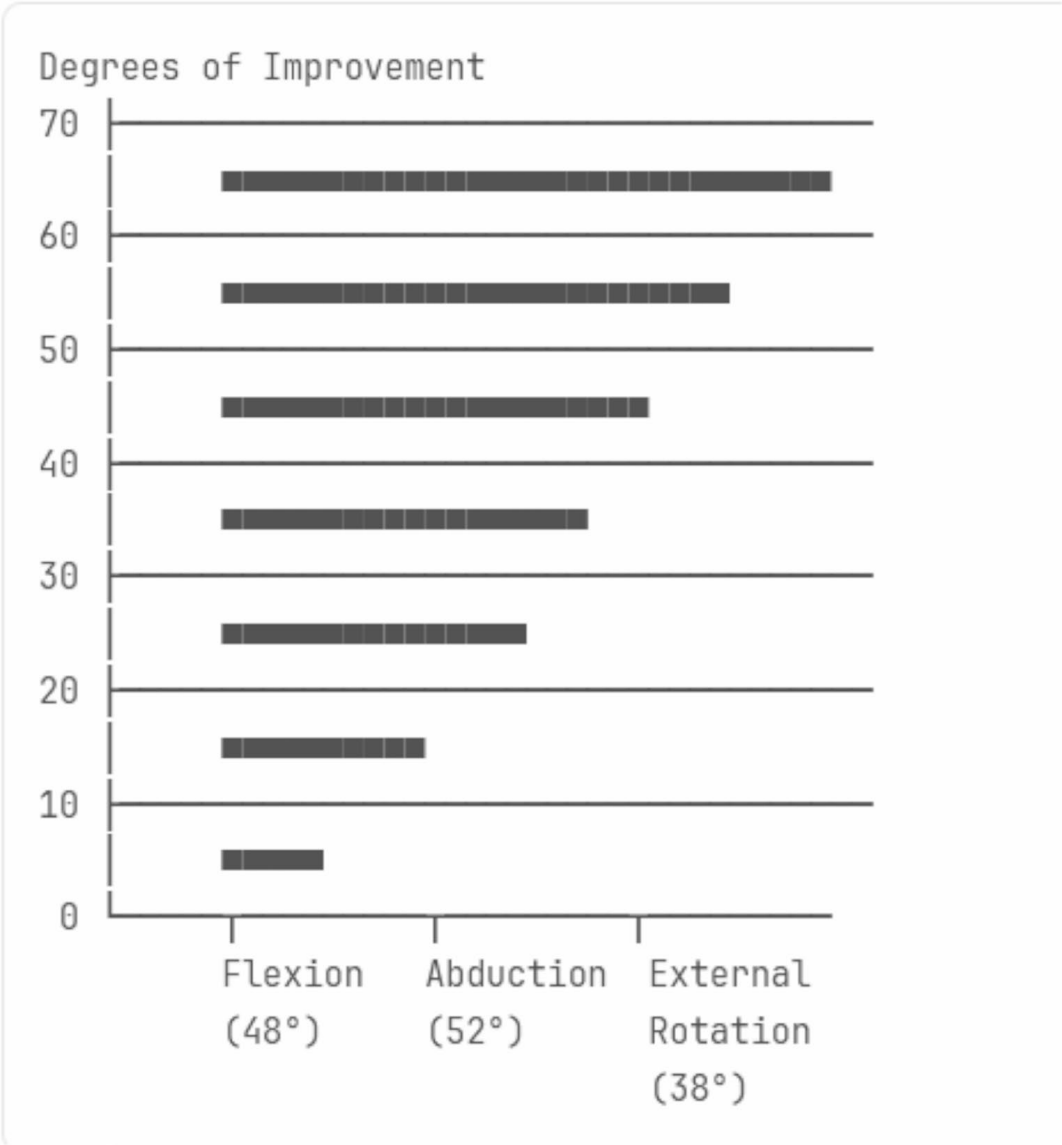
Range of motion improvement by movement type

**Fig. 3:**
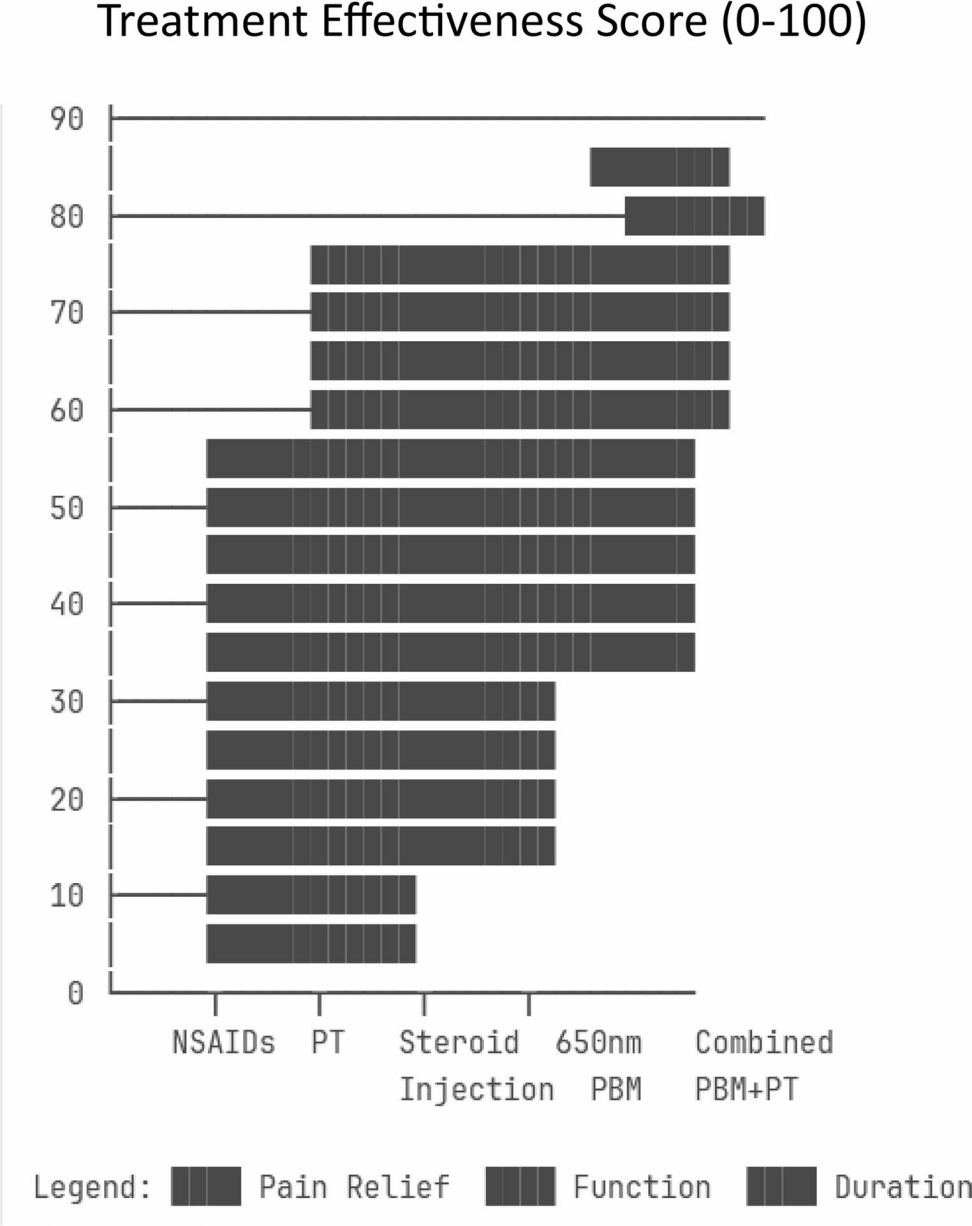
Comparative treatment effectiveness

## Discussion

### Summary of findings

This narrative review examines the current evidence regarding 650 nm diode laser photobiomodulation for frozen shoulder treatment. While laboratory studies demonstrate various cellular effects that could theoretically benefit tissue healing, the translation of these mechanisms to clinically meaningful improvements in frozen shoulder patients remains inadequately established.

The clinical evidence shows modest potential benefits, but is limited by small sample sizes, heterogeneous treatment protocols, significant risk of bias due to blinding challenges, and insufficient long-term follow-up. The variability in treatment parameters across studies makes it difficult to establish optimal protocols or predict treatment responses.

### Mechanistic understanding vs. clinical evidence gap

A critical limitation in the current literature is the tendency to extrapolate from well-established cellular mechanisms to clinical efficacy without sufficient direct evidence in frozen shoulder patients. Hamblin [9], Karu [10], and other investigators have provided robust evidence for mitochondrial mechanisms of photobiomodulation at the cellular level. However, several factors limit the clinical translation:

1. **Tissue Complexity:** In vivo tissues present complex interactions not replicated in cell culture
2. **Dosimetry Challenges:** Actual photon delivery to target tissues varies significantly from prescribed parameters
3. **Individual Variability:** Patient factors (skin pigmentation, tissue thickness, inflammation) affect light penetration
4. **Disease Heterogeneity:** Frozen shoulder presents varying pathophysiology across patients and disease stages

Henderson and Morries [23] addressed these complexity issues in their comprehensive approach to diagnosis and management of frozen shoulder, emphasizing the need to consider disease heterogeneity when implementing novel therapeutic approaches.

### Critical appraisal of clinical evidence

#### Methodological limitations

##### Blinding Challenges:

Most studies show high risk of bias for blinding due to the visible nature of 650 nm light. Sham devices have been used in some studies, but their adequacy for maintaining blinding is questionable. This limitation significantly affects interpretation of subjective outcomes like pain and function.

##### Placebo and Expectation Effects:

Laser therapy studies are particularly susceptible to placebo effects due to:

- High patient expectations for technological interventions
- Therapist enthusiasm and attention during treatment
- Difficulty in achieving adequate sham conditions
- Potential for inadvertent unblinding

##### Protocol Heterogeneity:

Significant variations in treatment parameters limit comparability:

- Power density variations (2–12 mW/cm^2^) may result in different biological responses
- Energy density differences (2–12 J/cm^2^) affect total photon dose
- Treatment duration and frequency variations impact cumulative effects
- Lack of standardized protocols prevents optimal parameter
  determination

#### Statistical and clinical significance

Many studies report statistically significant improvements that may not reach clinical significance thresholds:

- VAS pain reductions <2 points may not represent meaningful change
- ROM improvements <20° may not impact functional activities
- Short-term benefits without sustained long-term effects question
  clinical utility

### Multimodal treatment context

Contemporary evidence supports a comprehensive approach to frozen shoulder management. Kelley et al. [18] provided clinical practice guidelines emphasizing individualized, multimodal strategies. Page et al. [17] and Green et al. [20] established the efficacy of physical therapy and manual interventions as foundational treatments.

Recent reviews by [19, 22] emphasize that optimal outcomes require individualized, multimodal strategies that may include:

- Patient education and self-management strategies
- Graduated exercise programs and manual therapy
- Pharmacological interventions (NSAIDs, corticosteroids)
- Interventional procedures (intra-articular injections, hydrodilatation)
- Psychological support for chronic pain management

Within this context, PBM may serve as an adjunctive therapy rather than a primary treatment modality. The combination of PBM with exercise therapy shows promise in limited studies, suggesting potential synergistic effects that warrant further investigation.

### Special considerations for specific populations

#### Post-COVID-19 adhesive capsulitis

Macke et al. [24] reported emerging evidence suggesting that frozen shoulder associated with COVID-19 may have distinct characteristics requiring modified treatment approaches. Their investigation of ultrasound-guided infiltrative treatments combined with early rehabilitation showed promising results in this specific context, highlighting the importance of tailored treatment strategies for post-COVID adhesive capsulitis.

#### Diabetic patients

Diabetic patients with frozen shoulder often experience more severe symptoms and prolonged recovery. The potential anti-inflammatory effects of PBM may be particularly relevant in this population, though specific evidence remains limited. This population warrants targeted research investigation given the higher prevalence of adhesive capsulitis among diabetic individuals.

### Patient-centered outcomes and practical considerations

#### Patient adherence and tolerability

##### Limited data exists on:

- Treatment adherence rates across different protocols
- Patient preferences for PBM versus alternative treatments
- Factors influencing treatment completion
- Patient-reported experience measures

#### Cost-effectiveness and accessibility

##### Economic Considerations:

- Equipment costs: $5000–$25,000 for clinical-grade devices
- Training requirements for healthcare providers
- Treatment session costs: estimated $50–$150 per session
- Insurance coverage: variable and often limited

##### Accessibility Issues:

- Geographic availability of trained providers
- Equipment maintenance and calibration requirements
- Time commitment for multiple treatment sessions

### Limitations of current evidence

#### Study design limitations

- **Small Sample Sizes:** Most studies include <50 participants, limiting statistical power
- **Short Follow-up:** Few studies report outcomes beyond 6 months
- **Selection Bias:** Studies may preferentially include patients with specific characteristics
- **Publication Bias:** Negative studies may be underrepresented in published literature

#### Outcome measurement issues

- **Heterogeneous Outcome Measures:** Lack of standardized assessment tools
- **Timing Variability:** Different assessment timepoints across studies
- **Missing Data:** Incomplete outcome reporting in several studies
- **Minimal Important Differences:** Unclear clinical significance thresholds for many measures

#### External validity concerns

- **Population Generalizability:** Studies may not represent typical clinical populations
- **Setting Differences:** Research protocols may not reflect real-world clinical practice
- **Operator Dependency:** Treatment effectiveness may vary with provider experience

### Future research directions

#### Priority research questions

1. **Optimal Parameters:** Dose-response studies to establish standardized protocols
2. **Patient Selection:** Identification of patients most likely to benefit from PBM
3. **Combination Therapies:** Systematic evaluation of PBM with established treatments
4. **Long-term Outcomes:** Studies with follow-up periods ≥12 months
5. **Mechanism Validation:** In vivo studies confirming proposed cellular effects

#### Methodological improvements needed

- **Better Blinding:** Development of improved sham devices
- **Standardized Outcomes:** Consensus on core outcome measures
- **Larger Sample Sizes:** Adequately powered randomized controlled trials
- **Real-world Studies:** Pragmatic trials reflecting clinical practice

#### Economic evaluation

- **Cost-effectiveness analyses** comparing PBM to standard treatments
- **Budget impact studies** for healthcare system implementation
- **Quality-adjusted life year (QALY) assessments**

### Clinical implementation considerations

For clinicians considering PBM integration, Henderson and Morries [23] provide a comprehensive decision-making framework for adhesive capsulitis management that can incorporate photobiomodulation as an adjunctive modality.

#### Patient Selection Criteria:

- Patients seeking non-pharmacological alternatives
- Those with contraindications to corticosteroids
- Early-stage disease where conservative management is appropriate
- Patients willing to commit to multiple treatment sessions

#### Treatment Protocol Recommendations:

- Consider PBM as adjunctive to, not a replacement for, established treatments such as those recommended by [18]
- Ensure proper device calibration and maintenance
- Monitor for adverse effects and treatment response
- Maintain realistic patient expectations regarding outcomes
- Coordinate with physical therapy and other conservative interventions

#### Integration with Evidence-Based Protocols:

Bal et al. [15] and Ryans et al. [16] demonstrated that combining therapeutic modalities produces superior outcomes. Therefore, PBM should be considered within multimodal protocols rather than as monotherapy. Page et al. [17] and Green et al. [20] established exercise and manual therapy as foundational interventions, which should remain primary in any comprehensive treatment plan.

## Conclusions

Based on current evidence, 650 nm diode laser photobiomodulation shows potential as an adjunctive therapy for frozen shoulder management, but cannot be considered a validated standalone treatment. The existing clinical evidence, while suggesting possible benefits through mechanisms established by [9, 10], and other mechanistic researchers, is limited by methodological constraints, small sample sizes, and insufficient long-term follow-up.

The mechanistic rationale for PBM effects is compelling. Studies by Henderson and Morries [11] on mitochondrial redox signaling and [13] on mitochondrial dynamics have expanded our understanding of how photobiomodulation may influence cellular function. However, the gap between these laboratory findings and clinical outcomes remains significant.

Treatment effects, when present, appear modest and may not consistently reach clinically meaningful thresholds for all patients. Zhai et al. [8] systematically reviewed the evidence and similarly concluded that while some benefits are apparent, substantial heterogeneity limits definitive conclusions about efficacy.

Within the context of comprehensive frozen shoulder management, as recommended by [18, 19, 22], PBM may have a role as part of multimodal treatment strategies, particularly for patients seeking non-pharmacological alternatives or those with specific contraindications to other interventions. However, this application should be considered experimental pending higher-quality evidence from adequately powered, well-designed randomized controlled trials.

Healthcare providers considering PBM implementation should:

- Maintain evidence-based perspectives on its limitations, as outlined
  in systematic reviews by [8]
- Ensure patients have realistic expectations regarding potential benefits
- Continue established treatment approaches recommended by clinical practice guidelines [18]
- Position PBM as adjunctive therapy rather than monotherapy
- Consider patient preferences and treatment context when selecting modalities
- Participate in high-quality research efforts to advance the evidence base

The safety profile, as documented by [5, 6], is favorable for mild, transient adverse effects, making PBM a low-risk addition to multimodal protocols. However, evidence of superiority over established treatments remains lacking.

Future research priorities should focus on:

1. Standardizing treatment protocols to enable optimal parameter determination
2. Identifying optimal patient selection criteria to predict responders
3. Conducting adequately powered studies with meaningful long-term follow-up to establish true clinical utility
4. Developing improved mechanistic research linking cellular effects to clinical outcomes
5. Performing head-to-head comparisons with established treatments and multimodal approaches

The potential of 650 nm photobiomodulation therapy for frozen shoulder remains promising but currently unproven. With rigorous research addressing current evidence gaps and methodological limitations, future studies may establish a more definitive role for this therapeutic modality. Until such evidence emerges, PBM should be considered an experimental adjunctive therapy within comprehensive, evidence-based multimodal management strategies for adhesive capsulitis.
